# Transcriptomic analysis of submergence-tolerant and sensitive *Brachypodium distachyon* ecotypes reveals oxidative stress as a major tolerance factor

**DOI:** 10.1038/srep27686

**Published:** 2016-06-10

**Authors:** Irma Karla Rivera-Contreras, Teresa Zamora-Hernández, Ariana Arlene Huerta-Heredia, Jacqueline Capataz-Tafur, Blanca Estela Barrera-Figueroa, Piyada Juntawong, Julián Mario Peña-Castro

**Affiliations:** 1 Laboratorio de Biotecnología Vegetal, Instituto de Biotecnología, Universidad del Papaloapan, Tuxtepec, Oaxaca, México; 2 División de Estudios de Posgrado, Universidad del Papaloapan, Tuxtepec, Oaxaca, México; 3 Catedrática CONACyT-UNPA, Universidad del Papaloapan, Tuxtepec, Oaxaca, México; 4 Laboratorio de Cultivo de Células Vegetales, Instituto de Biotecnología, Universidad del Papaloapan, Tuxtepec, Oaxaca, Mexico; 5 Department of Genetics, Faculty of Science, Kasetsart University, Bangkok, Thailand

## Abstract

When excessive amounts of water accumulate around roots and aerial parts of plants, submergence stress occurs. To find the integrated mechanisms of tolerance, we used ecotypes of the monocot model plant *Brachypodium distachyon* to screen for genetic material with contrasting submergence tolerance. For this purpose, we used a set of previously studied drought sensitive/tolerant ecotypes and the knowledge that drought tolerance is positively associated with submergence stress. We decided to contrast aerial tissue transcriptomes of the ecotype Bd21 14-day-old plants as sensitive and ecotype Bd2-3 as tolerant after 2 days of stress under a long-day photoperiod. Gene ontology and the grouping of transcripts indicated that tolerant Bd2-3 differentially down-regulated *NITRATE REDUCTASE* and *ALTERNATIVE OXIDASE* under stress and constitutively up-regulated *HAEMOGLOBIN*, when compared with the sensitive ecotype, Bd21. These results suggested the removal of nitric oxide, a gaseous phytohormone and concomitant reactive oxygen species as a relevant tolerance determinant. Other mechanisms more active in tolerant Bd2-3 were the pathogen response, glyoxylate and tricarboxylic acid cycle integration, and acetate metabolism. This data set could be employed to design further studies on the basic science of plant tolerance to submergence stress and its biotechnological application in the development of submergence-tolerant crops.

Plants are genetically prepared to cope with soil flooding, as this is a challenge commonly faced during their lifespan. Flooding can be divided into two general categories: waterlogging, when water only covers the roots, and submergence, when even aerial tissues are left underwater^1^. In natural environments, flooding patterns have a strong influence on the type of species that colonize an area^2^. In agricultural fields, flooding can cause crop yield reductions that mount up to billions of dollars of losses^1^,^3^ and can cause a descendent economic cycle that can lead to poverty and migration^3^,^4^.Whether a consequence of days-long torrential rain associated with a tropical hurricane, or an hour-long winter rain, flooding can: disrupt O_2_ diffusion from the air to plant tissues, making O_2_ available at subnormal concentrations^5^ (<21% w/w, hypoxia); increase pathogen accession to cells^6^; block natural light^7^; and, when the water recedes, can create oxidative stress during recovery^8^. The systematic study of plant responses to waterlogging, submergence, hypoxic and anoxic stress has identified that plants take action towards diminishing the deleterious effects of these drawbacks. These responses also have normal physiological roles during germination^9^, organ development (reviewed in ref. 10), and crosstalk to other stresses, such as soil compaction, hydrocarbon pollution^11^, droughts, oxidative stresses^8^,^12^, the dark^13^ and pathogens^6^.

In plants, O_2_ supply disruption is confronted with a quick and sophisticated transition from an aerobic to anaerobic fermentative metabolism that can be divided into three phases: sensing, setting and maintenance. Oxygen sensing is achieved through constitutively expressed transcription factors of the *ETHYLENE RESPONSE FACTORS* group VII (*ERF*s-*VII*; reviewed in ref. 14), which possess a characteristic Met-Cys N-terminal domain^15^ (domain CMVII-1). Under normoxic (21% O_2_ w/w) conditions, ERFs-VII are substrates for the N-end rule enzymatic post-translational removal of Met^16^,^17^ and the concomitant oxidation of its second cysteine to cysteine sulfinic, or cysteine sulfonic acid by plant cysteine oxidases^18^ (PCO). This step occurs in the presence of O_2_ and the gaseous phytohormone nitric oxide (NO) and constitutes a signal for proteasome degradation^9^. Under hypoxic–anoxic conditions, cysteine oxidation is no longer performed and ERFs-VII proteins are stabilized and directed to the cell nuclei, where they induce transcriptional activation, directed towards setting an effective fermentative metabolism^16^,^17^. During this stage, a group of genes collectively known as the Hypoxia Core Genes (HCG) are induced; these include well-known fermentative enzymes such as alcohol dehydrogenase (ADH), pyruvate decarboxylase (PDC), alanine aminotransferase (AAT) and glutamic acid decarboxylase (GDH). HCG help to avoid the over-reduction of the NAD^+^ /NADH pool and maintain ATP synthesis through glycolysis and diverse fermentative routes at the expense of starch reserves^5^,^19^,^20^. This transcriptional activation is complemented with a translatomic change that has, as a consequence, the preferential ribosome loading of hundreds of signalling transcripts under anoxic conditions^21^.

During maintenance of the anaerobic metabolism, different auto-regulatory loops modulate the intensity of the response, of which two examples have been characterized: one involving *PCO* and the other the *HYPOXIA RESPONSE ATTENUATOR 1 (HRA1*). PCO promotes ERFs-VII cysteine oxidation and protein targeting to proteasomes^18^, while HRA1 inhibits ERFs-VII binding to DNA through direct protein–protein interactions^22^. Both *PCO* and *HRA1* are direct transcriptional targets of ERFs-VII. At the maintenance stage, a second group of strongly hypoxia-inducible ERFs-VII takes on the role of sustaining the transcriptional expression of HCG after the initial response^23^. If starch reserves are sufficient to fuel anaerobic energy extraction and allow survival until the water recedes, during reoxygenation, the plant cell has to manage a strong reactive oxygen species (ROS) burst, and paradoxically, a dehydration stress. Both challenges are faced with the induction of oxide-reduction enzymes and ABA sensitization^8^. Interestingly, at this stage, there is also another auto-regulatory loop that involves ERFs-VII, ABA and NO^9^.

The increased possibility of susceptibility to pathogen attack during submergence is counteracted with the expression of WRKY transcription factors that mobilize a toolbox of genes allowing the plant to recognize and act on bacteria and fungi, such as leucine-rich repeat kinases (LRRK), receptor-like kinases (RLK), wall-associated kinases (WAK) and peroxidases (PER)^6^.

The management of low light accessibility is achieved through a set of molecular events leading to increased angle (hyponastic) repositioning of the leaves outside the water level, thus raising the possibility of sustaining photosynthetic activity under stress^7^. Recently, it has been shown that the gaseous hormone ethylene, which accumulates around submerged tissues, coordinates cell expansion and division to promote the hyponastic response through the inhibition of *CYCLINA2;1* expression (*CYCA2*)^24^.

In addition to these general mechanisms – mostly discovered in the model plant *Arabidopsis thaliana* – plants that are native of flood-prone environments confront submergence with a set of physiological adaptations, such as aerenchymas, leaf gas films and focalized hyponastic responses^2^. However, most wild and agricultural relevant plants do not show these adaptations or have not developed them effectively.

The grass family (*Poaceae*) is one of the most important sources of products that satisfy human nutritional needs; cultivars of four domesticated grasses, namely maize (*Zea mays*; *Panicoideae*), wheat (*Triticum ssp*; *Pooideae*), sugarcane (*Saccharum ssp*.; *Panicoideae*) and rice (*Oryza sativa*; *Ehrhartoideae*), provide ≈50% of human caloric intake, with this proportion being even higher in developing countries^25^. However, molecular characterization of the mechanisms for tolerance to submergence in the grasses is uneven: in rice, this endeavour has been sustained for decades^26^, it has recently started in barley (*Hordeum vulgare*)^27^ and maize^28^ and only physiological characterization has been defined in wheat and sugarcane^29^,^30^. The natural genetic diversity of rice and maize has been used to find molecular mechanisms that are differentially expressed in tolerant versus sensitive varieties^28^,^31^.

Four loci associated with submergence tolerance have been characterized: *SUBMERGENCE1 (SUB1*), *SNORKEL (SK*) and *qAG-9-2* in rice and, more recently, *Subtol6* in maize. The genetic determinants of the *SUB1* and *SK* loci are ERFs-VII genes, *SUB1A* and *SK1/2,* respectively, and these orchestrate two different survival strategies^2^. *SUB1A* promotes the low-oxygen quiescence strategy (LOQS), which inhibits the gibberellic acid (GA) response, starch use, flowering and elongation to save energy resources while submerged^31^,^32^,^33^,^34^; and *SK1/2* orchestrates the low-oxygen escape strategy (LOES), which exacerbates GA signalling and promotes internode elongation, to escape submergence and reach the light^35^. *qAG-9-2* contains *TREHALOSE PHOSPHATE PHOSPHATASE 7*, a gene that permits submerged seed germination by altering the trehalose-6-phosphate (T6P)/sucrose ratio, with the consequence of promoting an accelerated anaerobic catabolism with amino acids as the final fermentative metabolites^36^. *Subtol6* is a maize locus that encompasses different submergence-induced genes, of which the most promising candidate as the main genetic determinant is *HAEMOGLOBIN 2 (HB2*) – a NO scavenger – that is constitutively expressed in tolerant cultivars and the expression of which is sustained under submergence^28^.

The discovery of the *SUB1* locus has allowed plant breeders in Asia to provide farmers with new non-transgenic varieties that have increased survival and yield rates after withstanding prolonged flooding^3^,^37^. *SUB1A-1* also improves drought tolerance^8^ as well as the quality of plant biomass as a raw material for biofuels^38^. Recently, the down-regulation of the N-terminal rule enzyme PROTEOLYSIS6 that destabilizes ERFs-VII proteins^14^ has been used to obtained barley varieties tolerant to waterlogging^27^. Research using the genetic diversity of *Arabidopsis*^39^, rice^40^ and aquatic plants^41^,^42^,^43^ has shown that there are other types of yet uncharacterized and species-particular molecular mechanisms that lead to improved submergence tolerance.

In this work, we employed ecotypes of the monocot model *Brachypodium distachyon*^44^ with different drought stress tolerances to screen for submergence-tolerant and submergence-sensitive variants. We then performed a comparative RNAseq-based transcriptomic study of these contrasting ecotypes under control and submergence stress in the presence of long-day photoperiod (16 h light/8 h dark). We hypothesized that the natural genetic diversity of this non-domesticated grass would allow us to find integrated mechanisms of tolerance.

## Results and Discussion

### Detection of *Brachypodium* ecotypes with contrasting tolerance to submergence stress tolerance. 

*Brachypodium* ecotypes have been previously screened for genetic material showing differential photosystem activity under drought stress^45^ and for contrasting flowering time and biomass architecture^46^. As Fukao *et al*.^8^ found that rice cultivars with enhanced submergence tolerance were also tolerant to drought stress; we decided to use this previous knowledge to select ecotypes that would display differential tolerance to submergence stress. We thus selected four ecotypes representing early or late flowering and high or low photosystem remnant activity under drought stress (Table 1).

Submergence stress has been applied to plants in different formats; for example, continuous dark^5^,^18^, continuous light^47^, a natural light cycle with midday harvesting^33^,^34^ and an artificial light/dark cycle with multiple collecting times^32^,^48^ or single collecting times^28^,^49^. Each approach has allowed the discovery of different submergence molecular responses, for example, lipid dynamics (continuous light)^47^, flowering inhibition (light/dark cycling)^32^ and dark-stress crosstalk (continuous dark)^5^.

In the present research, we decided to apply submergence stress under a controlled long-day (LD) light regime (16 h light/8 h dark) and to contrast the ecotypes tolerance at the juvenile (pre-flowering) stage. Our aim was to characterize a submergence response that included active circadian cycle oscillations^32^ and the management of light-dependent oxidative stress^5^.

**Figure 1:**
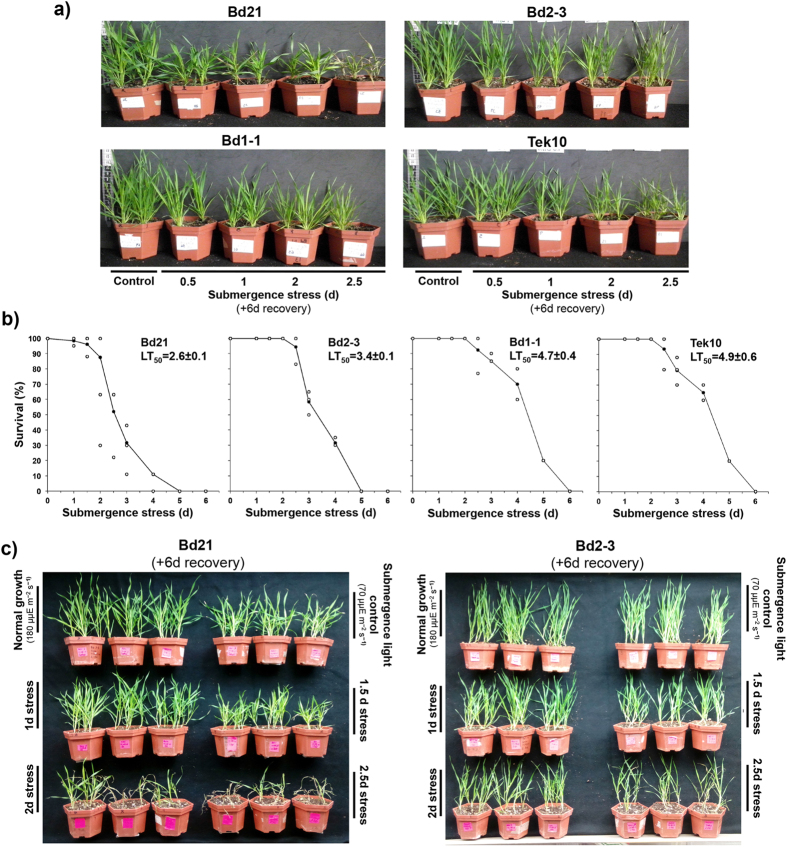
Survival of *Brachypodium distachyon* ecotypes after submergence stress. (**a**) Representative 20-day-old plants of each ecotype 6 d after being removed from the indicated submergence stress length. (**b**) Survival expressed as the median lethal time (LT_50_ ± S.D.) of each ecotype 6 d after being rescued from the indicated submergence stress. Each dot is an independent experiment. LT_50_ was calculated from three independent experiments in which all the ecotypes were present (n = 15 plants). (**c**) Phenotypic responses of the ecotypes selected for transcriptomic comparison (Bd21 and Bd2-3) to different lengths of time of submergence stress. Bd21 and Bd2-3 were considered as sensitive and tolerant ecotypes, respectively. Submergence light control and 2 d stress mRNAs were used for RNA-Seq and considered as the control (c) and stress (s) transcriptomes, respectively.

All four selected *Brachypodium* ecotypes expressed a quiescent submergence response, since their plant tissue did not elongate after submergence stress (Fig. 1a). Additionally, *Brachypodium* displayed known stress affectations, such as leaf death and stunted growth, when compared to controls grown side-by-side (Fig. 1a). This ecotype selection and format of submergence stress allowed the detection of contrasting tolerance material by both visual examination (Fig. 1a) and by median lethal time (LT_50_) quantification (Fig. 1b). Bd21 was the most sensitive ecotype (LT_50_ = 2.6 ± 0.1 d), while Bd2-3 was a moderately tolerant ecotype (LT_50_ = 3.4 ± 0.1 d) and Bd1-1 and Tek10 were the most tolerant ecotypes, with similar LT_50_ values (4.7 ± 0.4 and 4.9 ± 0.6 d, respectively). These data support the reports that drought and submergence stress share common tolerance mechanisms^8^, since the ecotypes with most tolerant photosystems to drought stress (Bd1-1 and Tek10; Table 1) were also the most tolerant under submergence stress.

### *Brachypodium* ecotypes with contrasting tolerance to submergence and transcriptomic analysis

To find the transcripts, pathways and mechanisms differentially expressed in the submergence-susceptible and submergence-tolerant ecotypes of *Brachypodium*, we used a sequence-by-synthesis differential gene expression approach (RNA-Seq). As submergence-sensitive ecotype we choose Bd21 and as its tolerant counterpart, we decided to compare with the most developmentally similar ecotype Bd2-3. Although Bd1-1 and Tek10 were the most tolerant ecotypes, they have a more extended juvenile stage, up to 4-5 months to flowering transition when compared to Bd21^46^. Bd21 and Bd2-3 have a closer developmental program and temporality in their juvenile stages (Table 1); still, they have a contrasting submergence tolerance by both visual and quantitative parameters (Fig. 1b,c). Highlighting the importance of comparing similar developmental stages, it has been shown that starch management is different in distinct developmental stages^50^ and that *SUB1A-1* had differential activities in the juvenile (pre-flowering) and mature (post-flowering) stages in *Arabidopsis*^38^. In order to use Bd1-1 and Tek10 as contrasting materials, we plan in the future to screen for submergence-sensitive material of similar developmental pattern.

In choosing the appropriate sample collection time, we considered the plants’ circadian cycles, since diurnal cycle and submergence stress have been jointly studied in few reports^32^,^48^. Based on the Zeitgeber Time (ZT0 = start of daylight), we decided to collect at ZT13 (3 h before the start of night). Our rationale was that this would allow us to incorporate in our data set diurnally oscillating transcripts that typically have low expression values^51^ and weak statistical significance at the more frequently used midday collection time, as previously shown for *FT* and *CONSTANS (CO*)^32^. We expect that these steps will increase the future usefulness of this data set to further characterize the importance of plants' diurnal cycle in the response to submergence stress.

The chosen intensity was 48 h after imposing the stress, since our survival data and visual examination of stress symptoms indicated that this was the earliest point where the survival outcome was different between Bd21 and Bd2-3 (Fig. 1b,c); therefore, it would allow us to find mechanisms active in sustaining survival. As controls, we decided to use plants growing inside empty tanks located to the side of the submerged plants, thus removing any effects of light stress (Fig. 1c).

**Figure 2:**
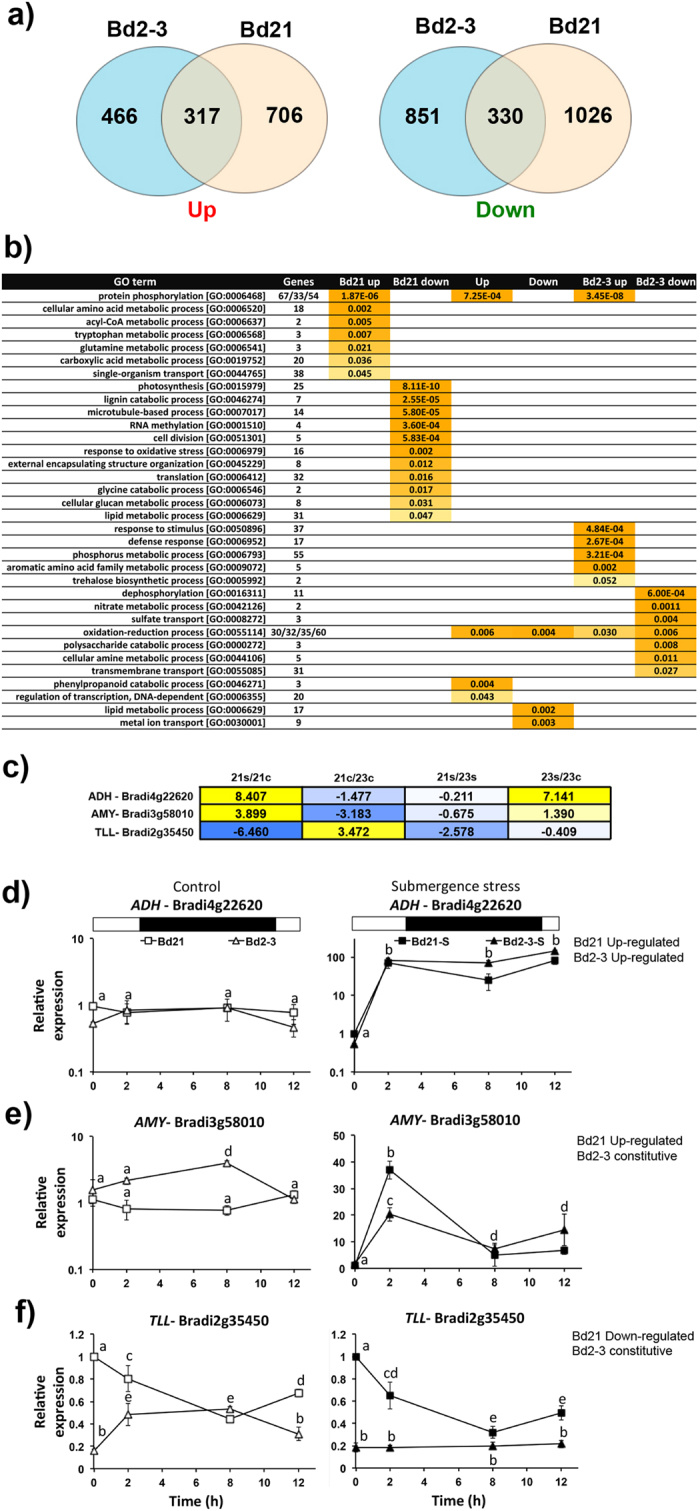
Transcriptome mobilization in the sensitive and tolerant ecotypes of *Brachypodium distachyon*. (**a**) Venn diagrams showing the number of commonly and exclusively up- and down-regulated transcripts in the ecotypes after 2 d submergence stress. Data obtained by RNA-Seq from three independent experiments (for each ecotype, n = 5 plants). (**b**) Gene ontology categories commonly and exclusively up- or down-regulated in the Bd21 and Bd2-3 ecotypes (p ≤ 0.05). (**c**) Log_2_FC of all four possible comparison categories of the selected transcripts representing three different groups to compare Bd21 and Bd2-3: Up–Up (commonly up-regulated; *ADH*), Up–Cons (up-regulated in Bd21, constant in Bd2-3; *AMY*) and Down–Cons (down-regulated in Bd21, constant in Bd2-3; *TLL*). (**d–f**) Transcript dynamics during the first 12 h of submergence stress, of *ADH* (**d**), *AMY* (**e**) and *TLL* (**f**). qPCR was used and the constitutive gene was *UBIQUITIN* (Bradi1g32860). Bd21 control at time 0 was selected as the relative expression (=1). The white and black symbols indicate control and submergence stress treatments, respectively. Data are the mean ± S.E. of three independent experiments with two technical repeats; letters indicate differences between the ecotypes and submergence times indicated (two-way ANOVA, P-value < 0.05).

A total of 2.91 × 10^8^ reads were obtained, with 1.4–3.1 × 10^7^ individual library reads and >97% positive mapping to the *Brachypodium* genome v 2.1 (Supplementary Table S1; GEO submission: GSE74222). To analyse the data, we selected an FDR of 5.0 × 10^−05^ and a Log_2_FC value >1.5 or <−1.5 for up- and down-regulated genes, respectively. There were 317 commonly up-regulated transcripts, 466 exclusively up-regulated in Bd2-3 and 706 exclusively up-regulated in Bd21. Regarding down-regulation, 330 transcripts were common, while 851 and 1026 were exclusive for Bd2-3 and Bd21, respectively (Fig. 2a). For abundance reference purposes, we looked for the most abundant up-regulated transcript in both ecotypes under submergence and it was Bradi4g44496, an unknown gene, with 8539 ± 689 counts per million (CPM); while for the control samples, it was Bradi4g45010, which codes for the protein asparagine synthase 1, with 9819 ± 1866 CPM (Supplementary Table S2).

With this information, we took three concomitant approaches to identify differentially expressed mechanisms. First, transcripts were classified into groups according to their differential expression among the two ecotypes (Bd21/Bd2-3), combining the three possible states of constitutively (Cons), up- or down-regulated; this resulted in eight groups (excluding constitutive–constitutive; Supplementary Table S2). This strategy was verified by fuzzy K-mean clustering analysis obtaining similar results (Supplementary Table S3). Second, a gene ontology (GO; Fig. 2b, Supplementary Table S4), an ortholog search (in *Arabidopsis* and rice) and PAGEMAN analysis of these transcript groups were performed (Supplementary Table S5, Supplementary Methods). Finally, a manual reconstruction of the known hypoxia pivotal biochemical routes was performed (Supplementary Fig. S1,2). As previously noted^5^,^19^, a significant proportion of the transcriptome corresponded to unknown/unannotated transcripts; this varied from 142 transcripts in the group preferentially down-regulated in Bd21, up to 246 transcripts in the up-regulated group in Bd21.

To observe the functionality of expression grouping and to investigate if our transcriptomic data could be useful to find genes with diurnal oscillatory expression, we chose one transcript to represent each group (Fig. 2c) and performed a dusk-dawn expression kinetics at ZT13, ZT15, ZT22 and ZT2, immediately after imposing the submergence stress (Fig. 2d). *ALCOHOL DEHYDROGENASE 1 (ADH1*, Bradi4g22620; group Up–Up), *α-AMYLASE 1 (AMY1*, Bradi3g58010; group Up–Cons) and *TRIACYLCLYCERLOL LIPASE LIKE 1 (TLL1,* Bradi2g35450; group Down–Cons) were selected. As expected from prior knowledge and from the transcriptomic data, *ADH1* was rapidly induced in both ecotypes on submergence and its expression was sustained during the stress period; however, while we observed an up-regulation in Bd2-3, it was not statistically significant. *AMY1* was significantly active and up-regulated at night in the Bd2-3 control plants; however, at dusk under submergence stress it was 2-fold more up-regulated in Bd21 than it was in Bd2-3; its highest expression peak also shifted 7 h earlier in both ecotypes (Fig. 2e). As observed in the RNA-Seq data, *TLL1* was down-regulated under submergence stress in Bd21, but was constant and irresponsive in Bd2-3. Interestingly in the controls, it was inversely regulated during night-time between the ecotypes (Fig. 2f).

These examples that the collection time used, near the end of the day (ZT13) when diurnally controlled transcripts have higher expression^51^, allowed us to capture in our transcriptomic data genes under circadian control that can be further characterized by expression kinetics. Most studies on submergence or hypoxic stress have not explored the role of the circadian cycle regulated genes, since they have applied stress in the dark as a standard condition^19^. When submergence was applied under a 10 h light / 14 h dark cycle, it was found that *HEADING DATE 3a (HD3a*) *–* the rice ortholog to the florigen gene *FLOWERING LOCUS T (FT*) – lost its circadian rhythmicity, leading to late flowering under stress, and that this effect was exacerbated by the rice *ERF-VII* gene *SUBMERGENCE1A-1 (SUB1A*), itself up-regulated at the end of the day under regular growth^32^. This ERFs-VII up-regulation at the end of the day on both normal and submergence conditions has also been reported in soybean^48^ (*Glycine max*) and has been observed in rice under dark stress^13^. The study of submergence stress under light conditions allowed involving very-long-chain fatty acids in a ROS regulatory role through acyl-CoA-binding proteins (ACBP), which are also capable of binding ERFs-VII proteins under normoxic conditions^47^.

### Oxidative stress management is differentially expressed in the submergence-tolerant ecotypes of *Brachypodium*

GO analysis showed that tolerant Bd2-3 significantly expressed both up- and down-regulated transcripts in the GO category oxidation–reduction processes (GO:0055114), while Bd21 had down-regulated transcripts in the GO process of the response to oxidative stress (GO:0006979; Supplementary Table S4; Fig. 2b). The 35 up-regulated transcripts in Bd2-3 included those that code for enzymes known to end-detoxify or use ROS^52^, such as ascorbate oxidases (ASO), ascorbate peroxidases (ASP), peroxidases (PER), p450 cytochromes (P450) and the Fe-S cluster biosynthetic protein frataxin (FRA)^53^.

**Figure 3:**
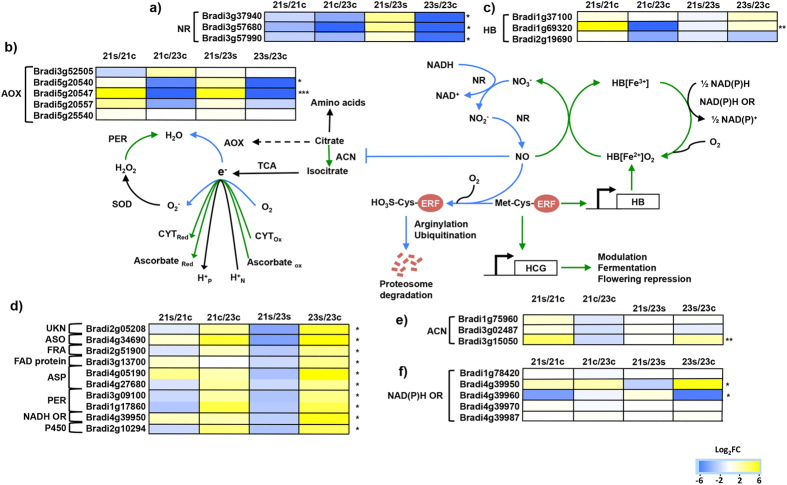
Integration of differentially expressed transcripts related to ROS management in tolerant and sensitive ecotypes of *Brachypodium distachyon* under submergence stress. (**a**) *NITRITE REDUCTASE* (NR). (**b**) *ALTERNATIVE OXIDASE* (AOX). (**c**) *HAEMOGLOBIN* (HB). (**d**) Transcripts involved in different ROS end-detoxification pathways: UKN (unknown), ASO (*ASPARTATE OXIDASE*), FRA (*FRATAXIN*), ASP (*ASCORBATE PEROXIDASE*), PER (*PEROXIDASE*). (**e**) *ACONITASE* (ACN). (**f**) NAD(P)H oxidoreductase (OR). Blue indicates down-regulation and yellow indicates up-regulation in Log_2_FC values after 48 h stress measured by RNA-Seq. The letters s and c indicate stress and controls, and the numbers 21 and 23 indicate Bd21 and Bd2-3, respectively. The green and blue arrows indicate up- and down-regulated activity in tolerant Bd2-3. *Significantly up- or down-regulated in Bd2-3,**significantly up- or down-regulated in Bd21,***significantly and inversely regulated in Bd2-3 and Bd21 (FDR < 0.05 × 10^−5^; Log_2_FC ≥ 1.5 or ≤ −1.5). Acronyms: SOD (superoxide dismutase), HCG (hypoxia core genes), TCA (tricarboxylic acid cycle), ERF (ethylene responsive factor), and NO (nitric oxide).

Transcripts involved in oxidation–reduction processes that were down-regulated in Bd2-3 could be subdivided into two further categories: those that, despite being down-regulated due to submergence stress in Bd2-3, still had the same expression when compared to Bd21 under stress; and those that were truly down-regulated in Bd2-3, compared to Bd21. In the first subset, we found isoforms of organic acids modifying enzymes (e.g. phosphoenlolpyruvate decarboxylase, aldehyde reductase and 6-phosphogluconate dehydrogenase), p450 cytochromes and ACC oxidases; these may indicate a stronger constitutive ethylene synthesis in Bd2-3 than in Bd21. In the second subset, we found more transcripts for p450 uncharacterized cytochromes and all three *Brachypodium NITRATE/NITRITE REDUCTASE* (NR) annotated genes (Bradi3g37940, Bradi3g57680, Bradi3g57990). *NRs* were expressed more in the control Bd2-3 than in Bd21 (combined transcript abundances of 760–913 and 90–307 CPM, respectively) and were more strongly down-regulated in Bd2-3 than in Bd21 by submergence stress (31.9–44.8 and 75.0–82.6 CPM; Log_2_FC −4.3 and −1.2, respectively; Fig. 3a). In this same category, we found two out of the five annotated *ALTERNATIVE OXIDASE (AOX*) transcripts (Bradi5g20540 and Bradi5g20547) differentially down-regulated in Bd2-3, when compared to Bd21 (constitutive or up-regulated; Fig. 3b).

The simultaneous down-regulation of transcripts for both ROS end-detoxification enzymes (AOX) and for ROS generating enzymes (NRs) in the tolerant ecotype Bd2-3 prompted us to look for the expression of components of the NO homeostasis cycle, especially transcripts coding for *HB1* and *NAD(P)H OXIDOREDUCTASES (NOR*)^54^. Three *HB*-like annotated genes were found: two of them were statistically constitutive in both control and stress plants for both ecotypes (Bradi1g37100, 28.1 ± 1 CPM; and Bradi2g19690, 1.22 ± 1.03 CPM) and a third transcript (Bradi1g69320, *HB1*), which was grouped with the Up–Cons genes because of its very low expression in the controls of Bd21 (0.14–0.19 CPM) and its constitutive expression in control Bd2-3 (26.2–69.2 CPM). Under submergence stress, *HB1* was strongly up-regulated in Bd21 but remained statistically constant in Bd2-3 (8.1 and 0.69 Log_2_FC, respectively; Fig. 3c). We found five annotated *NOR* genes, two of them were up- or down-regulated, but accounted only for 3% of global *NOR* transcripts, while the others were not differentially expressed among the ecotypes or treatments.

**Figure 4:**
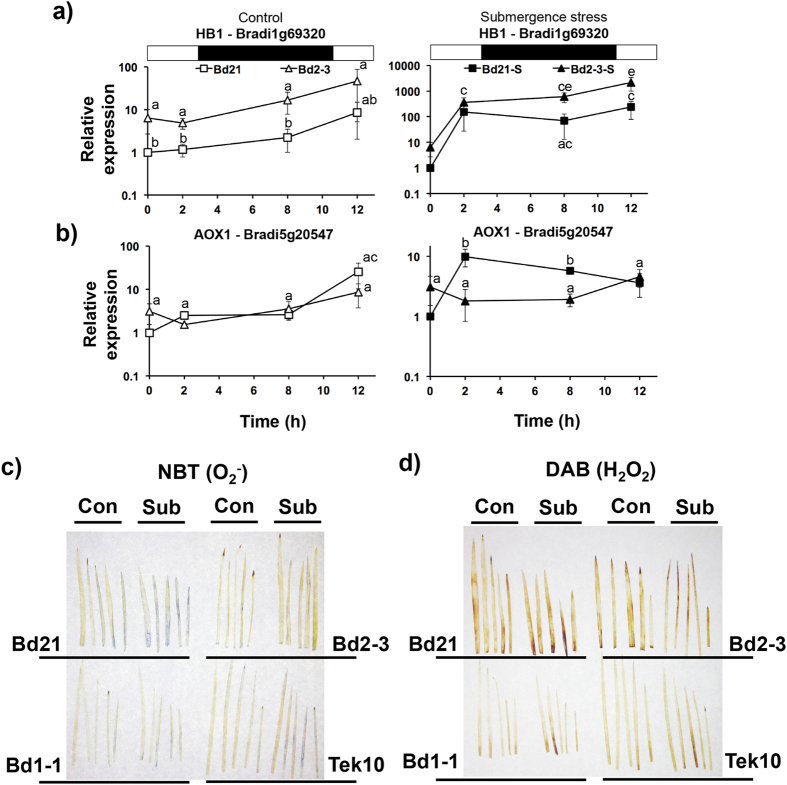
Expression of transcripts coding for ROS scavenging proteins and ROS detection in leaves of submergence-sensitive and submergence-tolerant ecotypes of *Brachypodium distachyon*. (**a,b**) Transcript dynamics during the first 12 h of submergence stress of *HB1* (**a**) and *AOX1* (**b**). qPCR was used and the constitutive gene was *UBIQUITIN* (Bradi1g32860). Bd21 control at time 0 was selected as the relative expression (=1). The white and black symbols indicate control and submergence stress treatments, respectively. Data are the mean ± S.E. of three independent experiments with two technical repeats; letters indicate differences between the ecotypes and submergence times indicated (two-way ANOVA, P-value < 0.05). (**c,d**) Leaves from the submergence stress sensitive (Bd21) and tolerant (Bd2-3, Bd1-1 and Tek10) ecotypes after 24 h submergence stress and the respective controls showing *in situ* presence of superoxide (**c**) and hydrogen peroxide (**d**), as a result of NBT and DAB staining, respectively.

We further measured *HB1* and *AOX1* expression by qPCR during the first 12 h of stress. As indicated by RNA-Seq, Bd2-3 had a higher pre-stress *HB1* expression than Bd21. The difference was maintained until the early morning (ZT12), when its expression also increased in Bd21, probably indicating a normal ROS burst after the restart of illumination, as previously proposed by Lee *et al*.^5^. When submergence stress was imposed, *HB1* was more expressed in Bd2-3 than in Bd21, especially late at night (ZT22) and in the early morning (Fig. 4a). *AOX1* expression under control conditions was low and remained constant throughout the night and until the morning, when a significant increase in expression was detected only in Bd21; under submergence stress, *AOX1* expression in Bd2-3 overlapped with that of the control, however in Bd21 it was up-regulated throughout the night (Fig. 4b). These data may indicate that Bd21 under submergence stress suffers early oxidative stress and relies on downstream ROS management enzymes, such as AOX, while Bd2-3 ROS homeostasis is efficiently managed through *HB1.*

To visually assess the significance of these molecular data at the physiological level, leaves of all four *Brachypodium* ecotypes studied were stained after 24 h of submergence stress with NBT (Fig. 4c) and DAB (Fig. 4d), indicating superoxide and peroxide presence, respectively 8,28. For both ROS, the submergence-sensitive Bd21 showed more staining than the more tolerant ecotypes. We also quantified formazan absorbance (superoxide) and Amplex Red oxidation (peroxide) after 24 h of submergence stress obtaining similar results (Supplementary Methods and Supplementary Fig. S3).

Taken together, these data suggest that an important determinant for submergence tolerance in these *Brachypodium* ecotypes is the coordinated management of oxidative stress, ranging from the attenuation of NO generation by *NR* down-regulation, NO scavenging through *HB1* and diversification of the final electron acceptor options, such as ascorbate and water (Fig. 3d). Interestingly, Campbell *et al*.^28^ also found a constitutively expressed *HB1* concomitant with the down-regulation of *AOX* in maize varieties with superior submergence tolerance, opening the possibility that this mechanism may be extended to agriculturally relevant grasses.

In pre-flowering plants, the control of ROS toxicity would not be the only benefit obtained from a robust ROS management system. From the knowledge obtained in *Arabidopsis,* it would also have, as a consequence, the stabilization of ERF-VII proteins through NO removal^9^ and, in turn, would improve stress sensing^14^,^16^,^17^, sustain the expression of HCG^23^, avoid tricarboxylic acid (TCA) cycle inhibition by NO at the aconitase step^55^ (Fig. 3e), and create a positive regulatory loop, since the promoter of *HB1* is a direct target of ERFs-VII^56^. These steps would improve plant survival, not only to hypoxia but also to other stresses too^12^.

One scenario where NO removal would be counterproductive for submergence tolerance is during underwater germination. In germinating rice, *HB-like* transcripts are repressed during submergence^57^; this would allow NO to accumulate and degrade ERFs-VII, thereby promoting germination^9^.

### Signalling, phosphorylation and pathogen response

**Figure 5:**
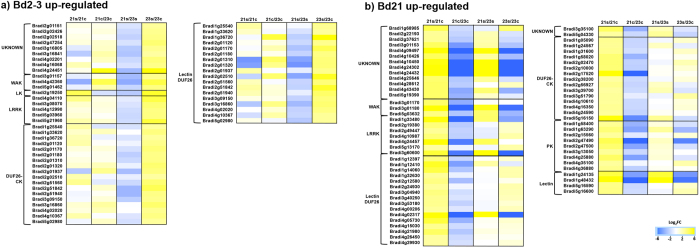
Differentially expressed transcripts coding for phosphorylation signalling cascades in sensitive and tolerant ecotypes of *Brachypodium distachyon*. (**a,b**) Blue indicates down-regulation and yellow indicates up-regulation in Log_2_FC values after 48 h stress, measured by RNA-Seq. Significantly (FDR < 0.05 × 10^−5^) up-regulated (Log_2_FC ≥ 1.5) transcripts in the tolerant Bd2-3 ecotype (**a**) and the sensitive Bd21 ecotype (**b**). Enzyme acronyms: WAK (wall-associated kinase), LK (light-repressible kinase), LRRK (leucine-rich repeat kinase), DUF26-CK (domain of unknown function 26 cysteine-kinase), and PK (protein kinase).

Transcripts involved in protein phosphorylation (GO:0006468) were commonly up-regulated in both Bd21 and Bd2-3 (33), as well as being differentially induced (67 and 54, respectively). The analysis of these transcripts showed a complex picture that involves proteins of a diverse nature, such as wall-associated kinases, leucine-rich-repeat kinases and light-repressible kinases, and proteins containing the domain of unknown function 26 of both kinase- (CK) and lectin-like families (DUF26; Fig. 5). We propose that a differential plant pathogen response may harmonize the detection of this transcripts group.

Submergence increases the bacterial load by an order of magnitude, when compared to controls, and submerged plants counteract this with a coordinated pathogen (fungi and bacteria) response that includes pattern recognition receptor proteins, most of them kinases^6^. Of special interest are the *DUF26* transcripts, which constitute half of this group in *Brachypodium* (Fig. 5). Miyakawa *et al*.^58^ concluded through structural and binding assays that GINKBILOBIN2 – a DUF26 lectin protein from *Ginko biloba* – acts as a specific mannan-binding protein that exerts an antifungal blocking of cell-wall biosynthesis. Supporting the role of DUF26 proteins during pathogen defence, Wrzaczek *et al*.^59^ found dynamic expression patterns of *DUF26* kinase transcripts in response to extracellular ROS induced by ozone that resembles a plant pathogen response. Interestingly, Narsai and Whelan^57^ performed a transcriptomic meta-analysis contrasting rice (representing a hypoxia tolerant genetic background) and *Arabidopsis* (hypoxia sensitive) at the germination stage; they found that *DUF26* transcripts were preferentially up-regulated in rice, probably preparing the seedling to thrive in an aqueous pathogen-prone environment. Our ortholog analysis indicated the same results in *Brachypodium*, highlighting the importance of DUF26 proteins in monocot response to submergence stress (Supplementary Table S5, ORTHOMCL1093).

Our transcriptomic data indicated that most of the transcripts (49 of 54) related to protein phosphorylation that were up-regulated in Bd2-3 under submergence stress remained constant in Bd21, or were not expressed to the same degree during stress, when compared to Bd2-3; conversely, most of those up-regulated in Bd21 under stress (51 of 67) were already constitutively expressed at the same level in the control Bd2-3, so did not need further up-regulation under stress in Bd2-3 (Fig. 5). With this information, we hypothesized that Bd2-3 is an ecotype constitutively prepared to detect and respond to a wider range of pathogen signals. In support of this hypothesis, Bd2-3 preferentially up-regulated transcripts involved in defence responses (GO:0006952). A further research step would be to cross-compare submergence tolerance to pathogen sensitivity.

### Differential changes in primary metabolism

**Figure 6:**
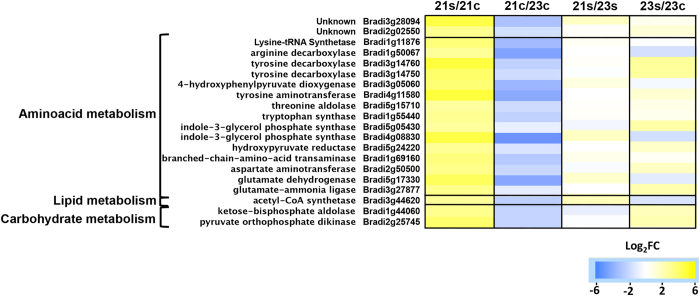
Transcripts grouped in carboxylic acid metabolic processes (GO:0019752) that were up-regulated in *Brachypodium distachyon* Bd21 ecotype. Significantly (FDR < 0.05 × 10^−5^) up-regulated (Log_2_FC ≥ 1.5) in sensitive Bd21 after 48 h of submergence.

Twenty transcripts coding for the carboxylic acid metabolic process (GO:0019752) were found to be up-regulated as Log_2_FC values during stress in Bd21 (Figs 2b and 6). These transcripts included *ASPARTATE AMINOTRANSFERASE 3* (Bradi2g50500, *ASP3*) and *PYRUVATE ORTHOPHOSPHATE DIKINASE* (Bradi2g25745, *PPDK*), both known to belong to the HCG in *Arabidopsis* and rice, respectively^5^,^60^ . However, differential expression values of these transcripts are the result of being less abundant in the Bd21 control plants than in Bd2-3; under submergence stress, these transcripts are equally abundant in both ecotypes (Fig. 6), suggesting that Bd2-3 is constitutively better prepared to face stressful conditions.

In this same category, there were transcripts for enzymes involved in aromatic acid metabolism, such as indole-3-glycerol phosphate synthase (Bradi5g05430, Bradi4g08830, I3GPS) and tryptophan synthase (Bradi1g55440, TRP). Nevertheless, other homologues were detected to be up-regulated in Bd2-3 (GO:0009072). Under submergence stress in rice, aromatic acids accumulate and can function as buffers for carbon conservation and are later consumed during recovery^61^,^62^. We decided to manually reconstruct the aromatic amino acid biosynthetic pathway and to take into account the transcripts for all the isoforms (Supplementary Fig. S1). Two of three transcripts coding for 3-deoxy-D-arabino-heptulosonate 7-phosphate synthase (DHAP synthase) – an enzyme that catalyses the first committed step for aromatic amino acid biosynthesis – were significantly up-regulated and more abundant in Bd2-3 under submergence stress. However, when all three transcripts were analysed, the Log_2_FC was below the 1.5 threshold value (1.09). Similar cases resulted from the quantification of transcripts for *I3GPS, CORISMATE SYNTHASE, TRYPTOPHAN SYNTHASE* and *PHENYLALANINE AMMONIA-LYASE*. The quantification of aromatic amino acids and the testing of inducible silencing of these transcripts should provide insights into the physiological significance of these expression differences and their routes under submergence stress.

Three categories of primary metabolism were down-regulated under stress in Bd21, compared to Bd2-3 (Fig. 2b), namely photosynthesis (GO:0015979), translation (GO:0006412) and the lipid metabolic process (GO:0006629); for the first two categories, these transcripts coded for light-harvesting complex and ribosomal proteins, respectively. Transcripts in the lipid metabolic process were a diverse group coding for desaturases, lipases and synthases. These three categories have been well documented as relevant for stress tolerance. SUB1 rice plants maintain higher chlorophyll contents than intolerant varieties^33^, while hypoxic stress disrupts ribosome integrity^21^,^63^ and lipid metabolism is selectively regulated under submergence stress^47^ and increased by *SUB1A-1*^*32*^. For all three categories, most of the transcripts were more abundant in the Bd21 control plants than in Bd2-3 and were equally expressed during stress, highlighting the importance of pre-stress constitutive biochemical capabilities.

We reconstructed the pyruvate fermentative pathways active in *Brachypodium* (Supplementary Fig. S2). Even though we could not detect differential expression under stress between the ecotypes, we found transcripts simultaneously up-regulated for all the reported routes, starting at carbohydrates and leading to ethanol, lactate, alanine and gamma-amino butyric acid^60^,^63^. We also found transcripts for all steps of the glyoxylate and TCA cycles were active, and even up-regulated, in the critical steps needed to allow them to work together (Supplementary Fig. S2); for example, *ACONITASE (ACN*)*, MALATE SYNTHASE (MLS*) and *ISOCITRATE LYASE (ICL*) were up-regulated. *ICL* was the 7th most abundant transcript under submergence stress (3799 ± 174 for Bd21 and 5616 ± 738 CPM for Bd2-3). This multi-organelle and multi-route integration has been previously proposed as pivotal for anaerobic germination in rice^64^, not only as a carbon conservation pathway, but also for aldehyde detoxification and as an antioxidant pathway, through the alternative “suicide protein” functions of abundant *ALDEHYDE DEHYDROGENASE* (Bradi4g31310, *ALDH*; 2699 ± 87 CPM). *ALDH* was constitutively more expressed in Bd2-3 controls (745 ± 170 and 1956 ± 405 CPM, for Bd21 and Bd2-3 respectively; Log_2_FC = −1.4 Bd21c/Bd23c). Our transcriptome study highlighted that the integration of the glyoxylate and TCA cycles is relevant in juvenile plants facing stress. This integration should be completed with the characterization of malate transporters; we found five annotated as malate:oxoglutarate antiporters, which were transcriptionally active (four marginally down-regulated) and one constitutively more abundant in Bd2-3 (Bradi4g33550; Log_2_FC = −1.6 Bd21c/Bd23c).

As our submergence experiments were performed under the influence of long-day illumination, we looked for transcripts annotated as coding proteins of the Calvin cycle (Supplementary Fig. S4), and found the pathway was active, emphasizing the importance of oxidative stress management derived from its activity. This was not surprising as Lee *et al*.^5^ demonstrated that the oxygen partial pressure increases rapidly after light appears at dawn. Interestingly, we observed that the most abundant *FRUCTOSE-1,6-BISPHOSPHATASE* transcript (Bradi2g24090, *FBP*) was strongly regulated in *Brachypodium* under submergence, up to the remarkable abundance of 2820 ± 319 CPM; in the context of the Calvin cycle, where *RUBISCO* transcripts were significantly down-regulated, but still present at a range of 525–803 CPM, *FBP* up-regulation would be a compensatory step under submergence stress.

### Flowering inhibition by submergence stress in *Brachypodium*

Flowering is inhibited by the ectopic expression of *SUB1A-1* in both rice^65^ and *Arabidopsis*, through the inhibition of *FT* and its rice ortholog *HD3a*^32^, this promotes starch conservation^38^. Interestingly, miRNA5200 targeting *FT-like* transcripts are also up-regulated under submergence stress in *Brachypodium*^66^. These temporary flowering delay mechanisms are proposed to be an energy-conservation feature of LOQS^32^. This has also been observed in field-grown rice subjected to submergence stress and is better modulated in tolerant SUB1 varieties^3^. In the *Brachypodium* Bd2-3 and Bd21 ecotypes, the heading date was delayed in proportion to the submergence intensity (Supplementary Fig. S5). Higgins *et al*.^67^ reconstructed the flowering pathways in *Brachypodium* and we used this knowledge to explore the *Brachypodium* flowering transcriptome. In addition to the previously found down-regulation of *CO* and *FT,* we also found the down-regulation of *HEME ACTIVATOR PROTEIN 5 (HAP5A*), a protein that aids in CO-induced *FT* transcription^67^ (Supplementary Fig. S5). However, no differences were detected among the ecotypes.

### ERFs-VII transcripts active under submergence stress in *Brachypodium*

Twenty transcription factors of diverse families (WRKY, ERFs, NACs, HSTF, ABI and ARFs) were commonly up-regulated in both ecotypes and grouped under the category of the DNA-dependent regulation of transcription (GO:0006355; Fig. 2b). Only WRKYs and ERFs have been previously characterized under submergence stress; the first orchestrate the pathogen response^6^ and the second control general HCG transcription^10^. Three were labelled as ERFs-VII (Bradi1g72457, Bradi2g11890 and Bradi2g27920). A BLASTP search of the N-terminal amino acids indicated that Bradi1g17960 (Bradi1g17961 in the V.2.0 *Brachypodium* genome) and Bradi1g72450 (not identified in RNA-Seq) were also ERF-VII transcripts.

**Figure 7:**
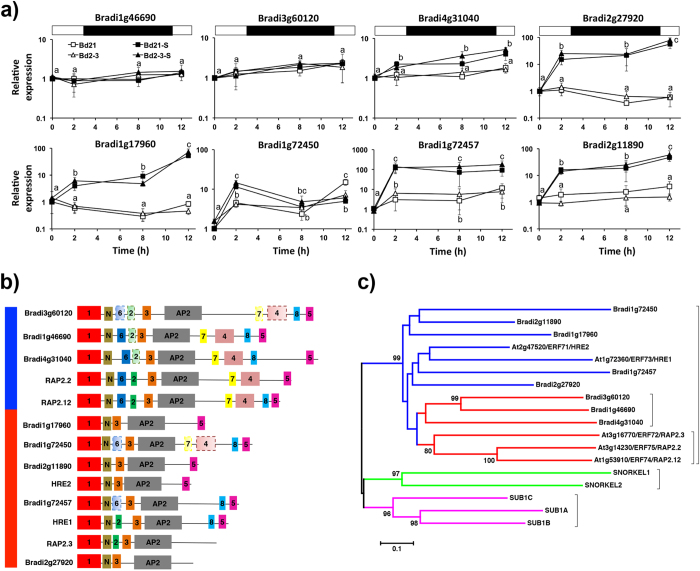
Characterization of *Brachypodium distachyon* group VII *ETHYLENE RESPONSIVE FACTORS* transcriptionally active during submergence stress. (**a**) Transcript dynamics during the first 12 h of submergence stress of ERFs-VII transcripts is indicated. qPCR was used and the constitutive gene was *UBIQUITIN* (Bradi1g32860). Bd21 control at time 0 was selected as the relative expression (=1). The white and black symbols indicate the control and submergence stress treatments, respectively. Data are the mean ± S.E. of three independent experiments with two technical repeats; letters indicate differences between the ecotypes and submergence times indicated (two-way ANOVA, P-value < 0.05). No statistical differences were detected among the ecotypes. (**b**) Domain architecture of *Brachypodium* ERFs-VII following previously published motifs^15^. The blue and red bars indicate constitutive and inducible expression during submergence stress. (**c**) Phylogenetic tree based on the amino acid sequence of *Arabidopsis, Brachypodium* and rice SUB1 and SNORKEL ERFs-VII. The blue and red bars indicate inducible and constitutive expression during submergence stress. The numbers are bootstrap values after 1000 replicates (≥80).

In *Arabidopsis*, ERFs-VII are divided into two categories: those up-regulated by hypoxic stress (HRE-like) and those constitutively expressed (RAP-like)^23^. N-terminal amino acids of RAP2.12 were used in a BLASTP search and we found that *Brachypodium* has three more ERFs-VII (Bradi3g60120, Bradi1g46690 and Bradi4g31040). The *Brachypodium* ERFs-VII family comprises in total eight genes (Fig. 7).

Since our RNA-Seq experiment was a 2-point collection data set, we quantified mRNA for these genes during the first night (12 h) of stress. In this analysis, we could not detect an ecotype-specific differential expression (Fig. 7a). This leaves the NO/HB-scavenging cycle as the most probable differential mechanism improving the ecotype tolerance in *Brachypodium* at the ERFs-VII protein level.

A manual and MEME-assisted analysis showed that this inducible/constitutive expression was correlated to a low/high domain diversity division (Fig. 7b), which could also be observed through phylogenetic analysis (Fig. 7c). Only Brad4g31040 – i.e. groups with constitutive ERFs – was still responsive to submergence stress, though not to the extent of inducible ERFs (Fig. 7a). We could not detect a SUB1-like ERF-VII in *Brachypodium* (Fig. 7c), confirming the ecological-niche uniqueness of this gene^68^,^69^. Knockout studies indicated that constitutive ERFs-VII work as oxygen sensors and early HCG inducers, while inducible ERFs-VII act as late-stress modulators^12^,^17^,^23^,^56^.

There is evidence that ERFs-VII respond to circadian rhythms and dark stress. SUB1A-1 ameliorates dark-stress damage^13^ and is expressed at the end of the night, independent of submergence stress^32^; this has also been observed for some ERFs-VII of soybean^48^. The analysis of *Arabidopsis* ERFs-VII transcripts in the DIURNAL transcriptomic database showed that *RAP2.3*, previously considered as a constitutive ERF, is actually highly responsive to the length of the day and has an expression peak at the end of the night. *RAP2.2* is less responsive, but still has an observable oscillation (Supplementary Fig. S6). In *Brachypodium*, we found that Bradi1g72450 was responsive to both submergence and night; interestingly, it was not the gene sharing the most domain types with RAP2.3 (being Bradi2g27920).

This ERFs-VII toolbox seems to be a common feature of all the plants analysed so far, both mono- and dicotyledonous^15^,^23^,^49^,^42^,^43^,^70^. Even though *Brachypodium* is not a plant characteristic of semi-aquatic habitats, small grasses can be subjected to natural submergence stress in the wild (Supplementary Fig. S7).

## Conclusions

Hypoxic stress responses are not only of importance for plant development but are also relevant during submergence stress and for the development of plant varieties tolerant to its negative effects. In this research, we aimed to discover physiologically relevant molecular mechanisms, by comparing the transcriptome of genetic materials with contrasting survival traits under submergence stress. By characterizing submergence-tolerant and submergence-sensitive ecotypes of the wild model grass *Brachypodium distachyon*, we found ROS management to be an important characteristic of the tolerant ecotype. This was most likely achieved through an integrated response involving constitutively expressed, induced and down-regulated mechanisms, such as the NO/*HB* cycle, general antioxidant responses and NR transcript expression, respectively. Transcripts involved in other mechanisms of increased complexity were also differentially expressed, for example transcripts of unknown function, signalling phosphorylation cascades (probably involved in the pathogen response) and the integration of the glyoxylate and TCA cycles. We expect that this information could be used to help design further experimentation aimed at expanding our current knowledge of physiological responses, with relevance for plant breeding programmes of submergence-tolerant crop cultivars.

## Methods

### Brachypodium ecotypes

Seeds of *Brachypodium distachyon* ecotypes Bd21, Bd2-3, Bd-1-1 and Tek10 were obtained from Professor David Garvin at the United States Department of Agriculture (USDA). For all the experiments, the seeds were disinfected in 10 mL of 1:1 household bleach (sodium hypoclorite 1.6%) and one volume of distilled, deionized and autoclaved water (ddH_2_0), rinsed five times in 20 mL of ddH_2_0, scarified in water for 4 d at 4 °C and sawn horizontally in substrate (Sunshine Mix #3 plus 1:4 v/v perlite:substrate, autoclaved for 2 h and finally mixed with 2% w/w slow liberation fertilizer NPK 15:15:17; Nitrofoska). Germination and growth were carried out under long-day conditions (16 h light/8 h dark, 180 μE m^−2^ s^−1^, 50% humidity) in a growth room, with irrigation every 2 d using filtered tap water.

### Submergence stress

*Brachypodium* plants (14-day-old, 6 leaves stage) were submerged in 30-cm deep water columns, inside opaque-walled plastic tanks. Light still reached the plants at 70 μE m^−2^ s^−1^. The ecotypes were submerged side-by-side in a randomized manner; only plants submerged in the same tank were compared. Controls were grown in plastic tanks without submergence. Submergence stress started at ZT13 (3 h before night) and the plants were removed by gentle subtraction from the water column and left to grow under normal conditions for the time indicated in each experiment. At the times indicated in each experiment, we registered the number of leaves, height, tillers, time to heading and percentage of surviving individuals; only the latter proved useful to quantify submergence stress tolerance, as 50% Lethal Time (LT_50_; using the on-line tool IC50; http://ic50.tk).

### RNA-Seq

*Brachypodium* ecotypes with contrasting submergence tolerance were subjected to a 48 h submergence stress, as detailed in the previous section. Above-ground tissue was collected immediately in liquid nitrogen and stored at −80 °C in an ultra-freezer, until further processing. Tissue was ground to powder with a mortar and pestle with liquid nitrogen, avoiding thawing. Control and submerged total RNA was extracted with TRIzol reagent (Invitrogen, 15596018), purified with Direct-zol RNA mini prep columns (Zymo Research, R2050) and digested in-column with DNAse I (ThermoScientific, #EN0521). RNA integrity and concentrations were verified in denaturing 1.0% agarose gel, a Nanodrop 2000 (ThermoScientific) and in a Bioanalyzer 2100 (Agilent), with the integrated software 2100 Expert. Samples had an RNA Integrity Number (RIN) between 6.4 and 7.2, characteristic of aerial plant tissue^71^. Total RNA extracted from the control and submerged tissue from three independent experiments, each consisting of five individual plants, were used to construct cDNA indexed libraries and sequenced in a HiSeq2500 (Illumina) at 1 × 50 format, making a total of 12 sequenced libraries (tolerant and intolerant ecotypes, control and submerged, experimental triplicates) in a 2-lane format. RNA integrity, library construction and sequencing were performed as a service at the Unidad Universitaria de Secuenciación Masiva, Instituto de Biotecnología, Universidad Nacional Autónoma de México (IBT-UNAM; http://www.uusmd.unam.mx).

### Bioinformatics analysis

Sequences were processed using the following pipelines. For base calling, Illumina Casava 1.7 software was employed and the sequenced reads were trimmed of their adaptor sequences using Trimmomatic^72^, and then mapped to the *Brachypodium distachyon* ecotype Bd21 genome (Bdistachyon_283_v2.0; downloaded from http://genome.jgi.doe.gov) using TopHat2 with parameters -p 4 —library-type fr-firststrand. Reads aligned to genomic regions were counted using the HTseq library^73^. The analyses of differentially expressed genes were performed in the R environment, using the edgeR package with a GLM (generalized linear model) and a false discovery rate <0.05 (FDR). Tables with CPM for all the gene models and results for the differential expression analysis were built (Supplementary Table S2). To group differentially expressed transcripts, a Logarithmic Fold Change (Log_2_FC) value ≥1.5 (up-regulated) or ≤−1.5 (down-regulated) and a FDR <0.05 × 10^−5^ were considered. GO analysis of these differential transcripts was performed at phytozome.jgi.doe.gov.

### Quantitative PCR

RNA was extracted from aerial tissue collected from five individuals of three new independent experiments with the Direct-zol RNA miniprep kit (Zymo Research, R2050) and digested with the included DNAse I in a column. Total RNA (2.1 μg) was used to synthesize cDNA using the Maxima First Strand cDNA kit (ThermoScientific, K1642). Quantitative PCR was performed in a Piko Real 96 thermocycler (ThermoScientific) using SYBR Green qPCR Master Mix 2× (ThermoScientific, K0251) and 1 μL of 1:10 cDNA dilution (20 μL final volume). Primers were designed using on-line tools at www.idt.com (IDT), with sequences downloaded from phytozome.jgi.doe.gov and synthesized by Macrogen. The efficiency was determined in 1:4, 1:6: 1:64 and 1:256 cDNA dilutions using the integrated thermocycler software (Supplementary Table S6).

### ROS staining

Samples were collected after 24 h of submergence stress. Two stain methods were used: nitrobluetrazolium (NBT) and 3,3′diaminobenzidine (DAB), detecting superoxide and hydrogen peroxide, respectively^8^. For NBT staining, the leaves were immersed in 25 mL of an NBT solution (0.5 μg/mL) in phosphate buffer (10 mM, pH 7.6) for 3 h in a rocking bed and protected from light. For DAB, the leaves were immersed in 25 mL of a DAB solution (1 μg/mL) in tris-acetate buffer (50 mM, pH 5.0) for 8 h in a rocking bed. After staining, the treatments were boiled in ethanol 95% (v/v) for 30 min; the ethanol was then decanted and the leaves were left immersed in glycerol (40%) in a rocking bed for 16 h and then photographed.

### Phylogenetic tree and domain analysis

Phylogenetic relations between the indicated ERFs-VII were performed with MEGA 6.0 software for the Mac^74^. Full-length amino acid coding regions were downloaded at NCBI from previous reports^23^,^33^,^35^, aligned using MUSCLE and then a phylogenetic tree was built by the neighbour-joining method (Poisson correction, pairwise deletion of gaps). Domain analysis was performed manually and MEME-assisted^75^ following the models published for *Arabidopsis*^15^.

## Additional Information

**Accession codes**: GEO submission GSE74222.

**How to cite this article**: Rivera-Contreras, I. K. *et al*. Transcriptomic analysis of submergence-tolerant and sensitive *Brachypodium distachyon* ecotypes reveals oxidative stress as a major tolerance factor. *Sci. Rep.* **6**, 27686; doi: 10.1038/srep27686 (2016).

## Supplementary Material

Supplementary Information

Supplementary Information

Supplementary Information

Supplementary Information

Supplementary Information

Supplementary Information

## Figures and Tables

**Table 1:** *Brachypodium distachyon* ecotypes studied and their phenotypic characteristics.

Ecotype	Photosystem activity under drought^a^	Leaf water content under drought^b^	Leaf biomass accumulation^c^	Flowering time^c^	Genotype class^c^	Submergence tolerance^d^
Bd21	Moderate	Moderate	Low	Early	1	Low
Bd2-3	Low	Low	Low	Medium	3	Moderate
Bd1-1	High	High	Moderate	Late	2	High
Tek10	High	High	High	Late	–	High

^a^Reported as Fv/Fm ratio^45^.

^b^Reported in^45^.

^c^Reported in^46^, except Tek^10^.

^d^Defined in this study.
