# Supplementary material for: Transcriptomic analysis of submergence-tolerant and sensitive Brachypodium distachyon ecotypes reveals oxidative stress as a major tolerance factor
Source: Sci Rep. 2016 Jun 10;6:27686. doi: 10.1038/srep27686 (PMC4901394; doi:10.1038/srep27686)
Supplement: Supplementary Information [file srep27686-s1.pdf]

Supplementary Figures, Legends, Tables, Methods and References

Transcriptomic analysis of submergence-tolerant and sensitive *Brachypodium distachyon* ecotypes reveals oxidative stress as a major tolerance factor.

Irma Karla Rivera-Contreras, Teresa Zamora-Hernández, Ariana Arlene Huerta-Heredia, Jacqueline Capataz-Tafur, Blanca Estela Barrera-Figueroa, Piyada Juntawong, Julián Mario Peña-Castro

Manuscript: SREP-16-02317A

Supplementary Figure S1

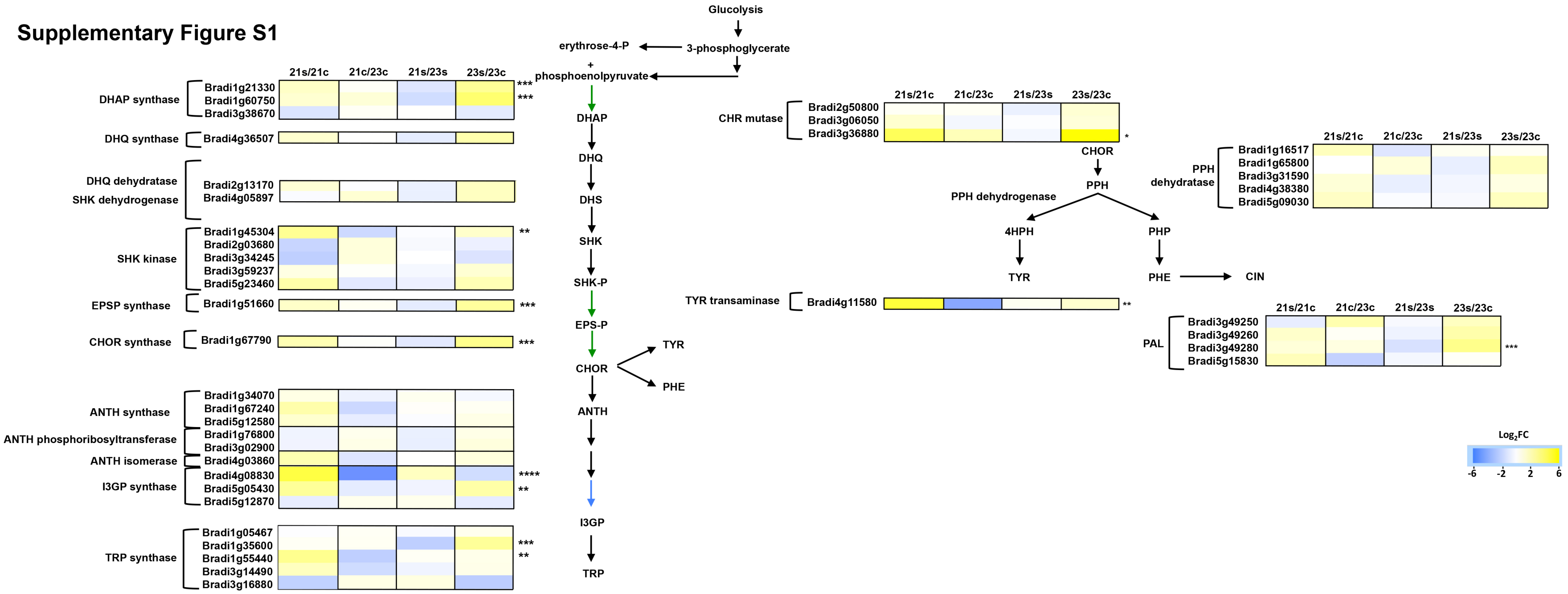

Supplementary Figure S2

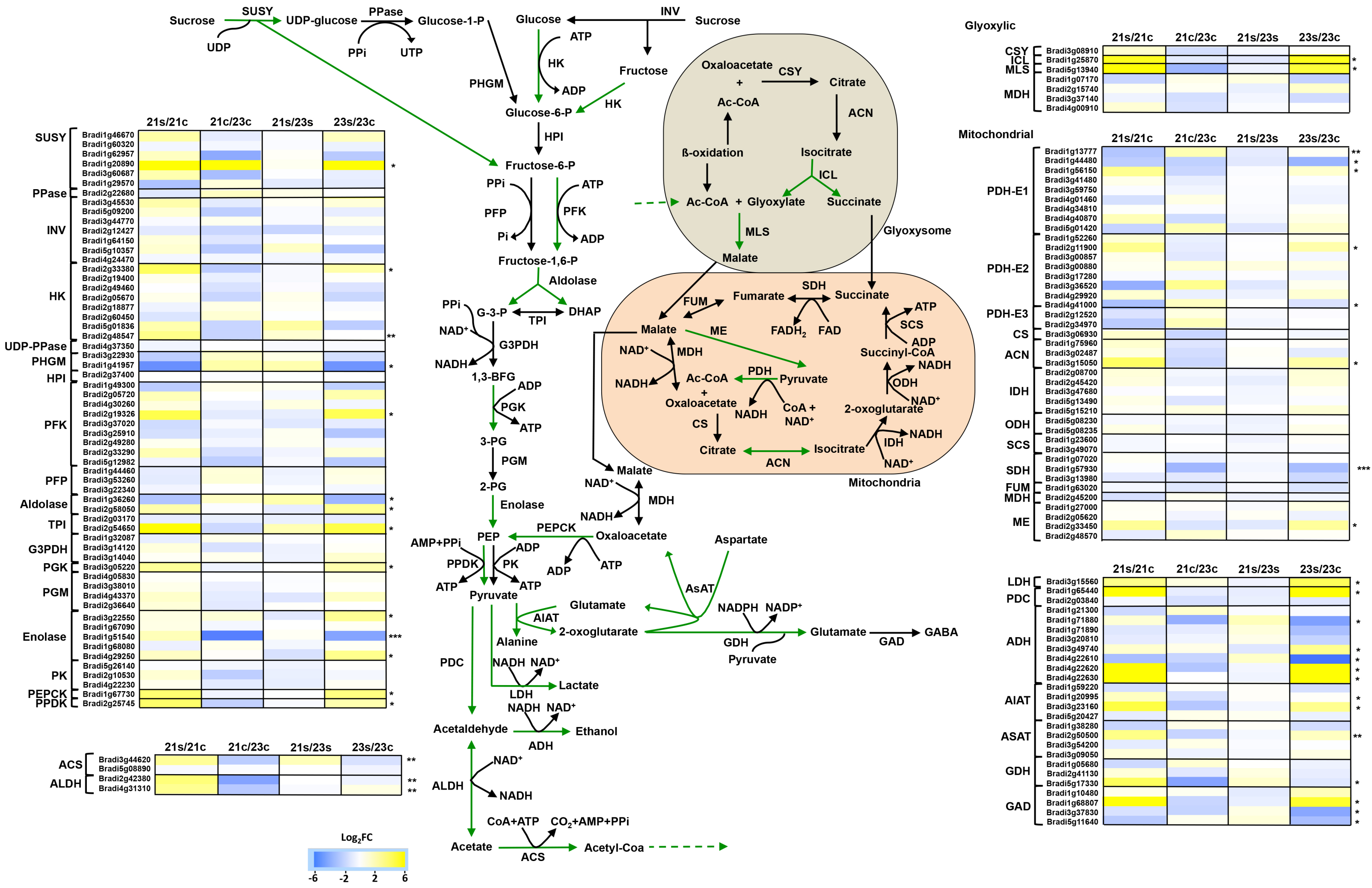

Supplementary Figure S3

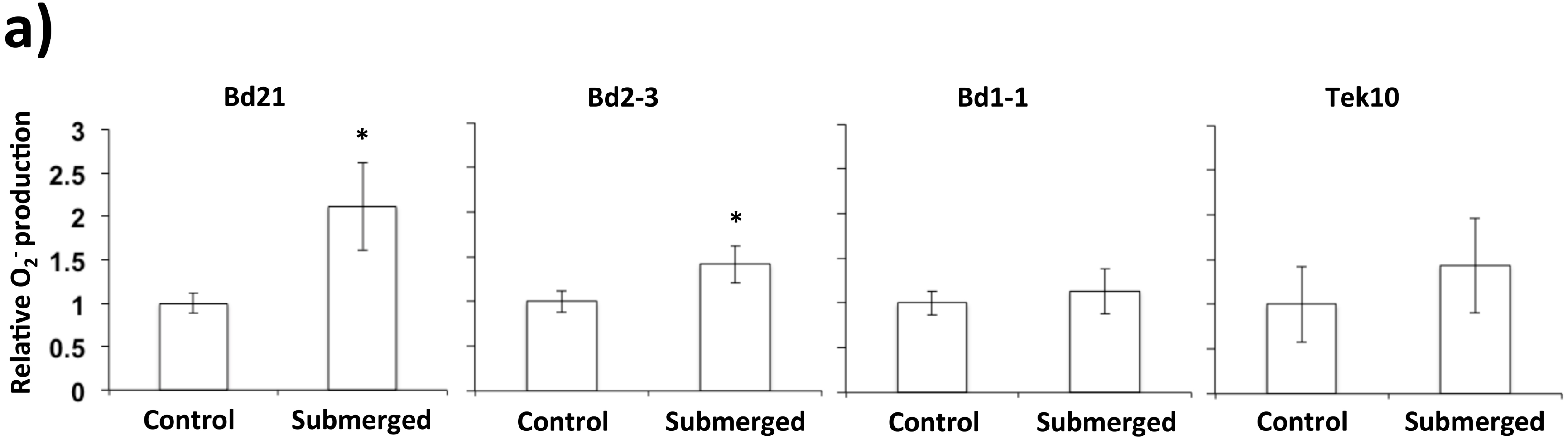

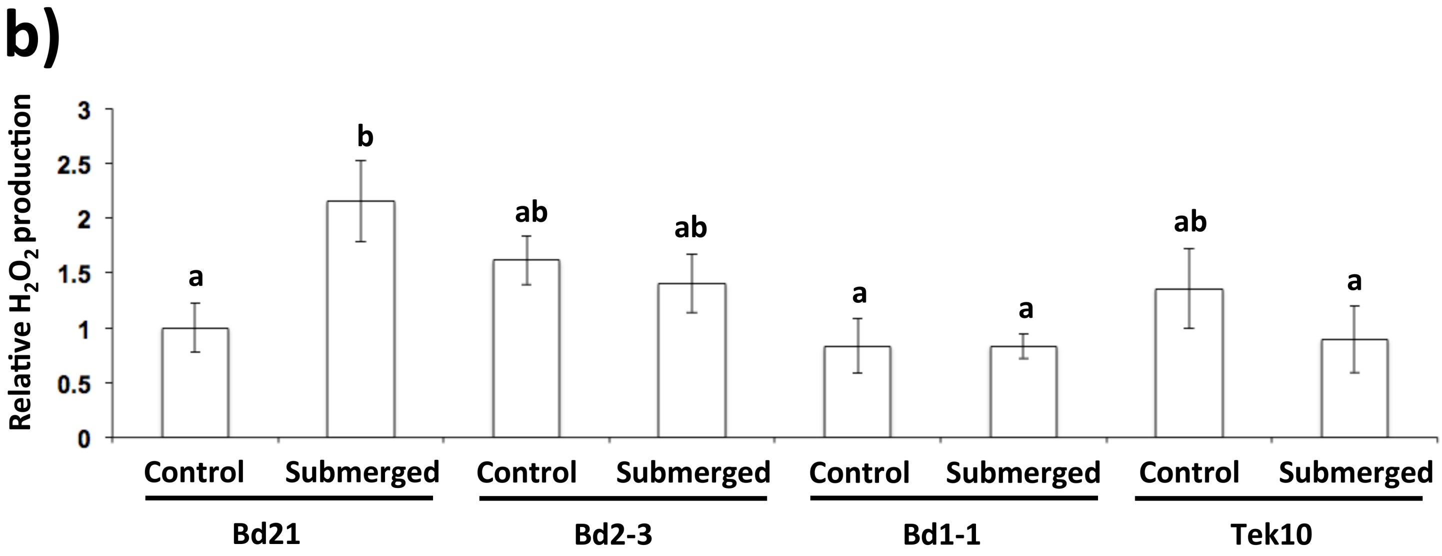

Supplementary Figure S4

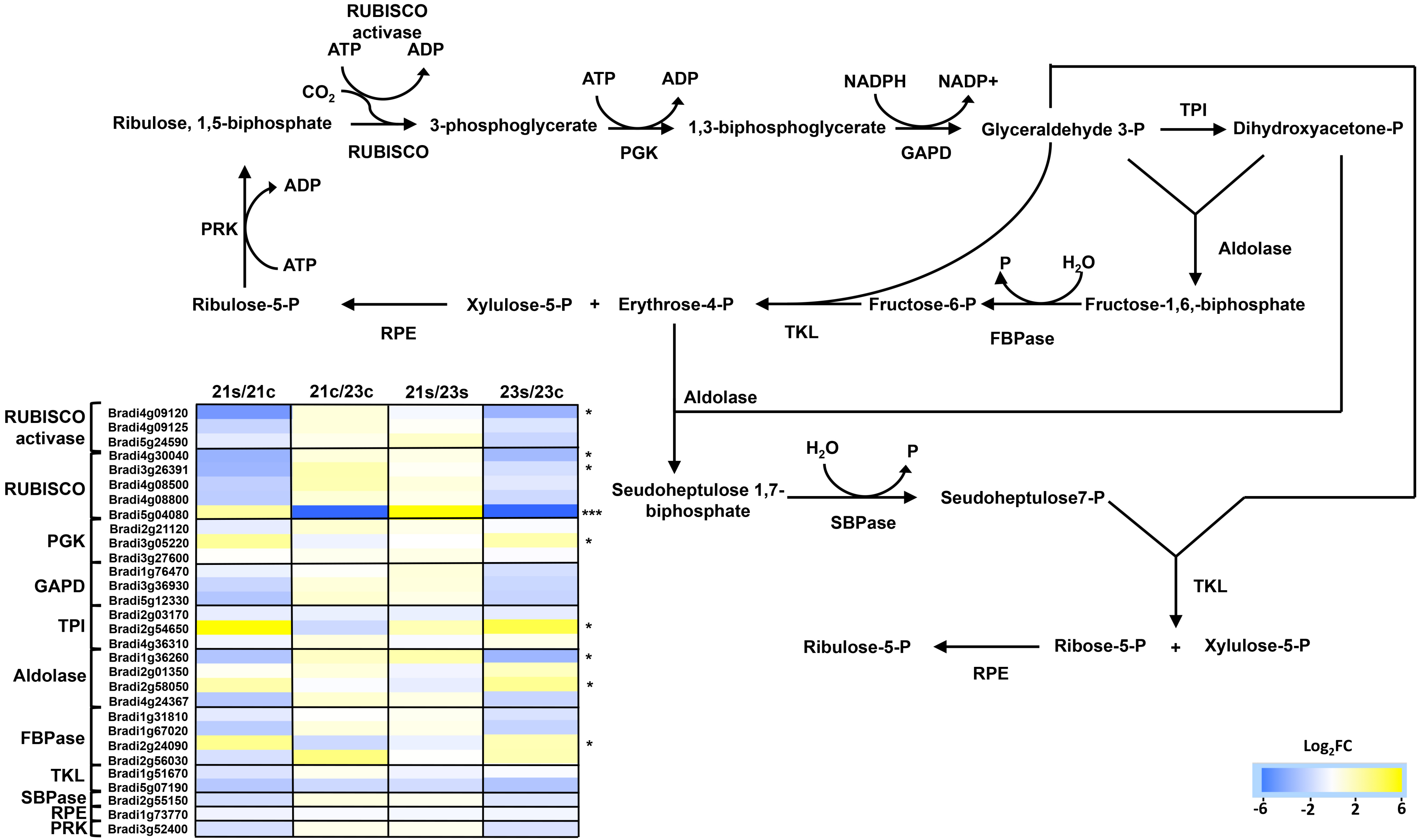

Supplementary Figure S5

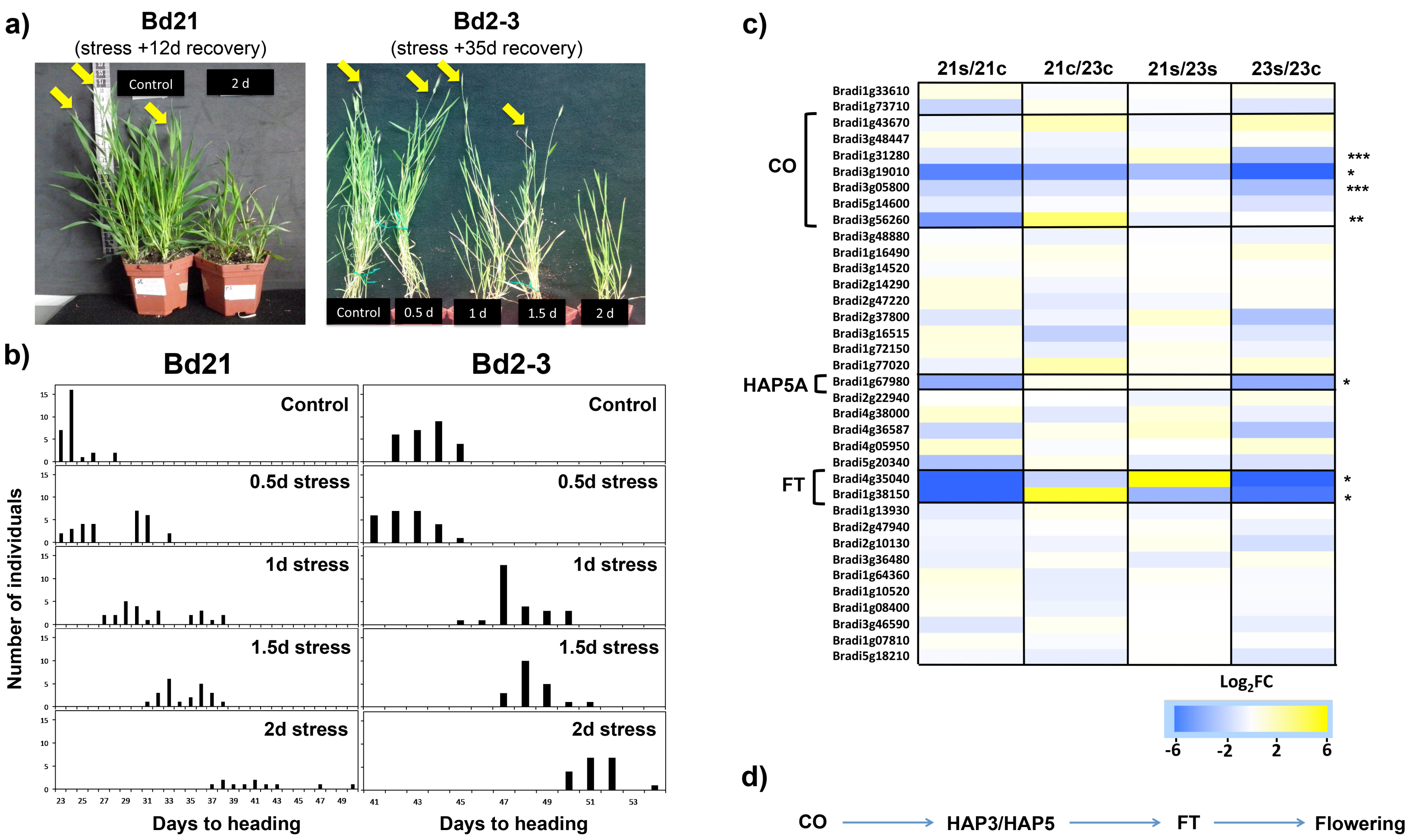

Supplementary Figure S6

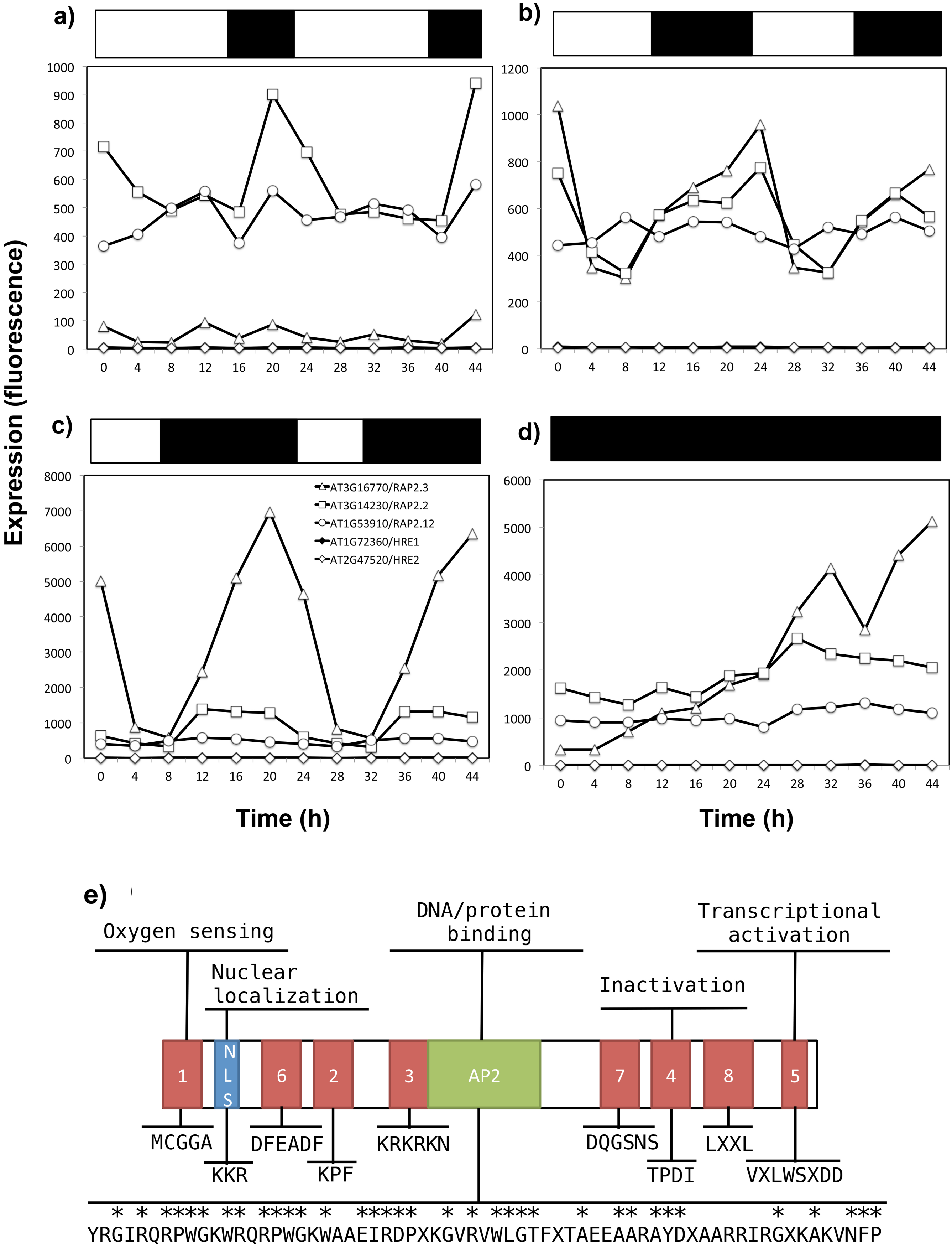

Supplementary Figure S7

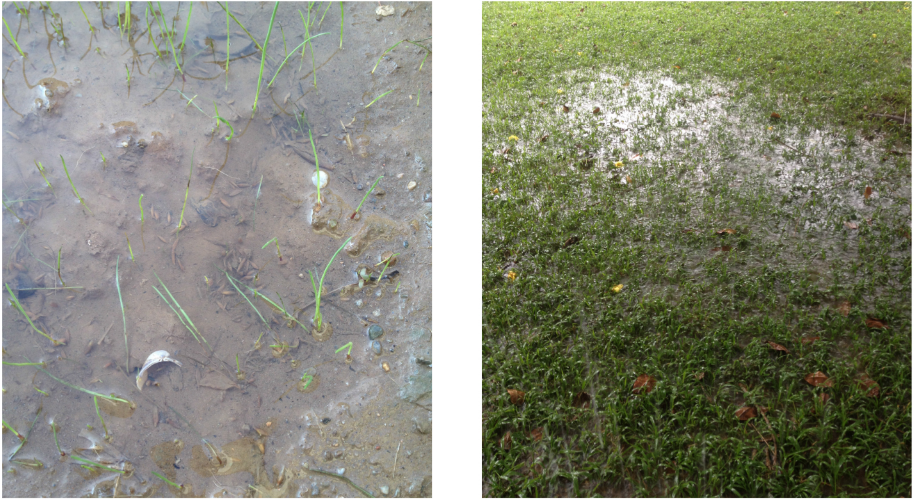

a) b)

Supplementary Figure Legends

Supplementary Fig. S1. Transcripts coding for the enzymes of the aromatic acids' (phenylalanine, tyrosine and tryptophan) biosynthetic pathways in submergence-tolerant and sensitive ecotypes of *Brachypodium distachyon* under submergence stress. Blue indicates down-regulation and yellow indicates up-regulation in Log_2FC values after 48 h stress, measured by RNA-Seq. The green and blue arrows indicate up- and down-regulated activity in tolerant Bd2-3. * Differential Log_2FC expression in both Bd21 and Bd2-3, ** differential Log_2FC expression in Bd21, *** differential Log_2FC expression in Bd23, **** significantly and inversely regulated in Bd2-3 and Bd21. Enzyme acronym: PAL (phenylalanine lyase). Metabolite acronyms: DHAP (3-deoxy-D-arabino-heptulosonate 7-phosphate), DHQ (3-dehydroquinate), DHS (3-dehydroshikimate), SHK (shikimate), SHK-P (shikimate phosphate), EPS-P (5-enolpyruvilshikimate-3-phophate), CHOR (chorismate), ANTH (anthrnilate), I3GP (indol-3-glycerol phosphate), PPH (prephenate), 4HPH (4-hydroxiphenylpyruvate), and PHP (phenylpyruvate).

Supplementary Fig. S2. Integration of transcripts coding for proteins of different fermentative pathways in *Brachypodium distachyon.* Blue indicates down-regulation and yellow indicates up-regulation in Log_2FC values after 48 h stress, measured by RNA-Seq. * Differential Log_2FC expression in both Bd21 and Bd2-3, ** differential Log_2FC expression in Bd21, *** differential Log_2FC expression in Bd23. The green and black arrows indicate up-regulation and irresponsiveness, respectively, under submergence stress in both ecotypes. Enzyme acronyms: SUSY (sucrose synthase), PPase (UDP-glucose pyrophosphorylase), INV (invertase), HK (hexose kinase), PHGM (phosphoglucomutase),

HPI (hexose-g-phosphate isomerase), PPi-PFK (pyrophosphate-dependent phosphofructokinase), TPI (triosephosphate isomerase), G3PDH (glyceraldehyde-3-phosphate dehydrogenase), PGK (phosphoglycerate kianse), PGM (phosphoglycerate mutase), PK (pyruvate kinase), PEPCK (phosphoenolpyruvate carboxykinase), PPDK (pyruvate orthophosphate dikinase), ACS (acetil-CoA synthetase), ALDH (aldehyde dehydrogenase), CSY (glyoxysomal or mitochondrial citrate synthase), ICL (isocitrate lyase), MLS (malate synthase), MDH (malate dehydrogenase), PDH (pyruvate dehydrogenase), ACN (aconitase), IDH (isocitrate dehydrogenase), ODH (2-oxoglutarate dehydrogenase), SCS (succinyl-CoA ligase), SDH (succinate dehydrogenase), FUM (fumarase), ME (malic enzyme), LDH (lactate dehydrogenase), PDC (pyruvate dehydrogenase), ADH (alcohol dehydrogenase), AlAT (alanine aminotransferase), AsAT (aspartate aminotransferase), GDH (glutamate dehydrogenase), GAD (glutamate decarboxylase). Metabolite acronyms: G-3-P (glyceraldehyde-3-phosphate), 1,3-BFG (1,3-biphosphoglycerate), 3-PG (3-phosphogycerate), 2-PG (2-phosphoglycerate), PEP (phosphoenolpyruvate), and GABA (ϒ-aminobutyric acid).

Supplementary Fig. S3. Superoxide and hydrogen peroxide production in aerial tissue of submergence-sensitive and submergence-tolerant ecotypes of *Brachypodium distachyon*. a) Superoxide detection after 24 h of submergence stress (ZT=2). Values were normalized to each ecotype control mean (± S.E.). Asterisk indicate significant differences between control and submerged treatments (Student's t test, $p<0.05$, n=3-6). b) H_2O_2 detection after 24 h of submergence stress (ZT=2). Values were normalized to Bd21 control samples (means ± S.E.). Letters indicate significantly different means (ANOVA, $p\leq0.05$, n=3-4).

Supplementary Fig. S4. Transcripts coding for Calvin cycle enzymes and expression in *Brachypodium distachyon* under submergence stress. Blue indicates down-regulation and yellow indicates up-regulation in Log_2FC values after 48 h stress measured by RNA-Seq. * Differential Log_2FC expression in both Bd21 and Bd2-3, *** significantly and inversely regulated in Bd2-3 and Bd21. Enzyme acronyms: RUBISCO (ribulose-1,5-bisphosphate carboxylase), PGK (phosphoglycerate kinase), GAPD (glyceraldehyde-3-phosphate dehydrogenase), TPI (triosephosphate isomerase), FBPase (fructose-1,6-bisphosphatase), TKL (transketolase), SBPase (sedoheptulose-bisphosphatase), RPE (ribulose-phosphate 3-epimerase), and PRK (phosphoribulokinase).

Supplementary Fig. S5. Flowering delay phenotypes of Bd21 and Bd2-3 and *Brachypodium distachyon* flowering transcriptome after 48 h of submergence stress.

a) Representative plants after the indicated times of stress and recovery. Yellow arrows point to the panicles.

b) Histograms showing the heading date of *Brachypodium* plants subjected to submergence stress for the indicated times. Data from two independent experiments (n = 13–17 plants).

c) Transcripts coding for different flowering pathways as published by Higgins *et al.*[67]. Blue indicates down-regulation and yellow indicates up-regulation in Log_2FC values after 48 h stress measured by RNA-Seq. * Differential Log_2FC expression in both Bd21 and Bd2-3, ** differential Log_2FC expression in Bd21, *** differential Log_2FC expression in Bd23. Protein acronyms: CO (constans), HAP5A (heme activator protein 5), and FT (flowering locus t).

d) Diagram showing the induction order of CO, HAP5A and FT.

Supplementary Fig. S6. Light-dependent expression of *Arabidopsis* ERFs-VII and the domain structure of RAP2.12.

A–D) Microarray expression signals downloaded from the on-line database DIURNAL (www.mocklerlab.org) for (A) Long-day (16 h light / 8 h dark), (B) balanced-day (12 h light / 12 h dark), (C) short-day (8 h light / 16 h dark), and (D) continuous darkness.

E) ERFs-VII domain structure[15] and reported activities[56]. CMVII-3 also has a high similarity to a nuclear localization signal.

Supplementary Fig. S7. Pictures showing in-field wild short grasses being submerged during a 30 min summer rain in the Gulf of Mexico (Tuxtepec, Oaxaca; August 2015).

A) Germinating seedlings.

B) Juvenile plants.

Supplementary Table S6. Primer sequences and qPCR parameters.

Gene	Name	Sequence	Tm (°C)	Product cDNA	Efficiency (%)	R^2
Bradi2g27920	Bradi920F	CTCTTGTAGCCTTGACTGTCG	62.1	116	102.2	0.99
	Bradi920R	TTATTTTGGATCAGAGGGTCCTG	62.1			
Bradi1g46690	Bd690-5F	CTACCACCTGTGATGGGTAATG	62	202	98.2	0.99
	Bd690-5R	GCACCAATAGACCTCAGATCC	62			
Bradi3g60120	Bradi120F	CACCAAGACTCCAGATACAACG	62.4	79	103.6	0.98
	Bradi120R	ACCCTTCACAACCGCATATTC	62.7			
Bradi2g11890	Bradi890F	ATGGCGTACGAGAACTACATG	61.8	147	97.7	0.99
	Bradi890R	GGACACTGATTTCTGCCACTAG	62.5			
Bradi4g31040	Bradi040F	GGCAAGTAATGTGGATGTCTG	61	150<br>250 (gDNA)	107.4	0.98
	Bradi040R	AACCATAGCGGATATAACCTGC	62.1			
Bradi1g17960	Bradi960F	ATCAGCAGCAACAGGAGG	61.6	82	120.2	0.97
	Bradi960R	ACCACTAATTACATAGACACGGC	62.2			
Bradi1g72450	Bradi450F	GTTCGTGTAAATGCAGAGCG	61.5	83	93.6	0.99
	Bradi450R	CCGATCCAAGGAAAAGGGAAG	62.6			
Bradi1g72457	Bradi457F	TTCTCTGCTATTACTGCCGC	61.9	81	103.8	0.99
	Bradi457R	CACGTCGAAATATGGCATTGG	61.8			
Bradi1g32860	UBI-860F	ACTTGCTTCTGTCTGGGTTC	62	203	103.5	0.99
	UBI-860R	GTAGAATTACACACGGGCTCA	62			
Bradi1g69320	HMB_F	ACGCCGTGTCTGTCTTTATC	62	128	98.0	0.98
	HMB_R	ATCTGCCACGCCGTATTT	62			
Bradi5g20547	AOX_F	GAGTCTTCTTCAACGCCTACTT	62	100	109.3	0.99
	AOX_R	GTATAAGACTTCACGGCCTCTTC	62			
Bradi2g35450	TRIGLI_F	CGTCGAGGATGTCGTTATCAA	62	96	103.5	0.97
	TRIGLI_R	CGGTATGCCAGTAGTCGTTATT	62			
Bradi4g22620	ADH2_F	GAGTACACCGTGATCCATGTC	62	104	102.8	0.99
	ADH2_R	GCACCAAGACCAGTTGAAATAC	62			
Bradi3g58010	AMY_F	GGAGCCCATGCATCTTCTATG	62	108	109.3	0.99
	AMY_R	GCTTGCTGTCCTTGTGGAT	62			

Supplementary Methods

Hydrogen peroxide and superoxide detection.

Samples were submerged for 24 h as described in Methods (except that were collected at ZT2), immediately frozen in liquid nitrogen, and ground to powder with a mortar and pestle. H_2O_2 was extracted in 200µl of 0.1% TCA (w/v) for 5 min, centrifuged at 10,000 g at 4°C for 10 min and the clarified supernatant was neutralized with 0.2M NH_4OH to pH 7-8 (Vera-Reyes *et al.*, 2015). Five µl were analysed using the Amplex Red hydrogen peroxide/peroxidase assay kit (A22188, Invitrogen) following the manufacturer's instructions. Reads were obtained at 570 nm in a Nanodrop 2000 spectrophotometer. Absorbance was normalized to dry weight of analysed biomass.

Superoxide was measured following the protocol of Grellet and Díaz-Ricci (2011). Briefly, plants were stained with NBT as previously described and lyophilized for 24 h or until constant weight. Plants were ground to powder with a mortar and pestle and formazan (product of NBT reduction) was extracted with 300 µl of 2M KOH and chloroform in a 1:1 ratio three times. Under dim light, chloroform was evaporated with a gaseous nitrogen flux and formazan was dissolved in 350 µl of DMSO and 300 µl 2M KOH at room temperature and immediately read at 630 nm in an UV/VIS spectrophotometer (Optizen Pop, Mecasys). Absorbance was normalized to dry weight of analysed biomass.

Fuzzy K-mean clustering and gene ontology enrichment analysis

Cluster analysis was performed on selected DEGs using Fuzzy k-mean clustering with Euclidean correlation for the distance measure, a membership exponent of 1.1, maximal number of iterations of 5000 and 20 clusters (Mustroph *et al*. 2009). For visualisation, the median Log_2FC values for each cluster were calculated. Genes in each cluster were analysed for gene ontology (GO) enrichment with the use of GOHyperGall Function (Horan *et al*. 2008). *B. distachyon* GO annotations were downloaded from phytozome.jgi.doe.gov.

Ortholog identification

ORTHOMCL version 1.4 (Li *et al*., 2003) was used to identify orthologs among *Brachypodium*, *Arabidopsis* and rice. The inflation number for this analysis was set to 1.2. Protein sequences used in this analysis were downloaded from JGI (Bdistachyon_283_v2.1.protein_primaryTranscriptOnly.fa.gz, Athaliana_167_TAIR10.protein_primaryTranscriptOnly.fa.gz, and Osativa_204_protein_primaryTranscriptOnl y.fa.gz).

PAGEMAN analysis

Brachypodium protein sequences were analysed using the Mercator pipeline to generate a mapping file (Lohse *et al*., 2013). *Arabidopsis* and rice mapping files were downloaded directly from http://mapman.gabipd.org/web/guest/mapmanstore. PageMan (Usadel *et al*., 2006) was used to visualise the overview of comparative transcriptome analysis. This over-representative analysis was analysed by Fisher's exact test with a cut-off value of two.

Supplementary References

Grellet, C. F. & Díaz-Ricci J. C. Quantitative determination of superoxide in plant leaves using a modified NBT staining method. *Phytochem. Anal*. **22**, 268-271 (2011).

Horan, K., *et al.,* Annotating genes of known and unknown function by large-scale coexpression analysis. *Plant Physiol*. **147**, 41-57 (2008).

Li, L., Stoeckert, C. J. & Roos, D. S. OrthoMCL: identification of ortholog groups for eukaryotic genomes. *Genome Res*. **13**, 2178–2189 (2003).

Lohse, M., *et al*. Mercator: a fast and simple web server for genome scale functional annotation of plant sequence data. *Plant Cell Environ*. **37**, 1250–1258 (2014).

Mustroph, A., *et al.* Profiling translatomes of discrete cell populations resolves altered cellular priorities during hypoxia in *Arabidopsis*. *Proc. Natl. Acad. Sci. USA,* **106**, 18843-18848 (2009).

Usadel, B., *et al*. PageMan: an interactive ontology tool to generate, display, and annotate overview graphs for profiling experiments. *BMC Bioinformatics* **7**:535 (2006).

Vera-Reyes, I., *et al*. Monoterpenoid indole alkaloids and phenols are required antioxidants in glutathione depleted *Uncaria tomentosa* root cultures. *Front. Environ. Sci.* **3**:27 (2015).
